# Bad Blood: Navigating VTE Risk in Breast, Ovarian, and Endometrial Cancer

**DOI:** 10.3390/cancers18162668

**Published:** 2026-08-18

**Authors:** Felice Sorrentino, Laura Vona, Luigi Nappi, Maria Rosaria Campitiello, Victoria Bitsadze, Jamilya Khizroeva, Alexander Makatsariya, Concetta Panebianco, Elvira Grandone

**Affiliations:** 1 Department of Medical and Surgical Sciences, Institute of Obstetrics and Gynecology, University of Foggia, 71122 Foggia, Italy; felice.sorrentino@unifg.it (F.S.); laura.vona@unifg.it (L.V.); luigi.nappi@unifg.it (L.N.); mr.campitiello@sanita.it (M.R.C.); 2 Department of Obstetrics, Gynecology and Perinatal Medicine, I.M. Sechenov First Moscow State Medical University, (Sechenov University), Trubetskaya Str. 8-2, 119435 Moscow, Russia; vikabits@mail.ru (V.B.); totu1@yandex.ru (J.K.); gemostasis@mail.ru (A.M.); 3 Thrombosis and Hemostasis Unit, Fondazione IRCCS Casa Sollievo della Sofferenza, 71013 San Giovanni Rotondo, Italy; c.panebianco@operapadrepio.it

**Keywords:** cancer-associated thrombosis, venous thromboembolism, breast cancer, ovarian cancer, endometrial cancer, hormones, thromboprophylaxis, endocrine therapy, immune-thrombosis

## Abstract

Cancer-associated thrombosis (CAT) is defined as venous or arterial thrombotic events occurring in patients with active malignancy or during anticancer treatment, most commonly presenting as venous thromboembolism and representing a leading cause of morbidity and mortality in oncology. Hormone-sensitive malignancies, including breast, ovarian, and endometrial cancers, are characterized by a complex interaction among tumor biology, endocrine signaling, inflammation, endothelial dysfunction, and coagulation activation. In these malignancies, venous thromboembolism (VTE) risk is influenced not only by intrinsic tumor-related mechanisms but also by patient-specific factors and anticancer therapies, particularly endocrine treatments, chemotherapy, targeted agents, and extensive surgical procedures. Breast cancer is generally associated with an intermediate thrombotic risk, although endocrine therapy—especially tamoxifen—significantly increases VTE incidence. Ovarian cancer represents one of the most thrombogenic solid tumors because of elevated tissue factor expression, inflammatory activation, advanced-stage presentation, and aggressive multimodal treatment strategies. Endometrial cancer exhibits a strong association with obesity, metabolic syndrome, prolonged estrogen exposure, and perioperative thrombotic complications. Emerging evidence highlights the role of immune-thrombosis, extracellular vesicles, inflammatory cytokines, and sex-specific coagulation pathways in cancer-associated hypercoagulability. Current guidelines increasingly support individualized thromboprophylaxis based on tumor biology, patient-specific risk factors, and bleeding risk assessment. This review summarizes the biological mechanisms linking hormones and thrombosis in breast and gynecologic cancers, discusses current evidence regarding VTE risk factors and treatment-related thrombotic complications, and explores modern approaches to biomarkers, risk stratification, and personalized thromboprophylaxis.

## Simple Summary

Cancer-associated thrombosis (CAT) is one of the most frequent and life-threatening complications in patients with gynecologic cancers, significantly contributing to morbidity, mortality, treatment interruptions, and increased healthcare costs. Breast, ovarian, and endometrial cancers display distinct thrombotic profiles driven by endocrine signaling, tumor biology, systemic inflammation, obesity, and anticancer treatments. In breast cancer, thrombotic risk is largely influenced by endocrine therapies, particularly tamoxifen. Ovarian cancer is among the most thrombogenic solid tumors, reflecting aggressive disease behavior, pronounced inflammatory activation, and intensive multimodal treatment approaches. Endometrial cancer is strongly linked to obesity-related inflammation, metabolic dysfunction, and perioperative thrombotic events. Understanding these sex-specific and tumor-specific mechanisms is crucial for accurate risk stratification and individualized thromboprophylaxis. With improving cancer survival, prevention of thrombotic complications is becoming an essential component of long-term oncologic care. Advances in biomarkers, molecular profiling, and artificial intelligence-based predictive tools are expected to further enhance personalized prevention strategies and improve clinical outcomes in patients with hormone-sensitive malignancies.

## 1. Introduction

Cancer-associated thrombosis (CAT) is defined as the development of venous or arterial thrombotic events in patients with active cancer. The pathogenesis of CAT is multifactorial and involves a dynamic interaction among tumor cells, endothelial dysfunction, inflammatory activation, platelet aggregation, and coagulation cascade activation [1]. Tumor cells directly promote thrombin generation through tissue factor (TF) expression, release of procoagulant extracellular vesicles, cytokine-mediated endothelial activation, and induction of neutrophil extracellular traps (NETs), all contributing to a hypercoagulable state [2,3]. Beyond tumor biology itself, anticancer therapies also contribute substantially to thrombotic burden. Chemotherapy, endocrine therapy, targeted agents, antiangiogenic drugs, surgery, central venous catheters, prolonged immobilization, and hospitalization all amplify thrombogenic risk [4]. Venous thromboembolism (VTE), including deep vein thrombosis and pulmonary embolism, occurs approximately four- to seven-fold more frequently in patients with malignancy than in the general population, whereas arterial thromboembolism occurs approximately twice as often [5,6,7]. It is a major cause of morbidity and mortality in oncologic patients and represents the second leading cause of death among patients with cancer after disease progression itself [8].

Hormones exert profound effects on vascular biology and hemostasis. Estrogens regulate coagulation factor synthesis, fibrinolysis, endothelial permeability, inflammatory signaling, and platelet activation, promoting a prothrombotic phenotype under several physiological and pathological conditions [9,10]. Consequently, hormone-sensitive malignancies—including breast, ovarian, and endometrial cancers—represent paradigmatic models for understanding the interaction between endocrine signaling and thrombosis. Thrombotic risk differs substantially among breast, ovarian, and endometrial cancers because of differences in tumor biology, inflammatory activation, treatment exposure, and patient comorbidities. Ovarian cancer, for example, exhibits one of the highest thrombotic burdens among solid tumors, whereas breast cancer generally carries intermediate risk, largely modified by endocrine therapies and metastatic disease [11].

As oncologic survival continues to improve, prevention and management of thrombotic complications have become increasingly important components of comprehensive cancer care and survivorship-oriented medicine. Personalized thromboprophylaxis strategies are therefore emerging as a crucial aspect of modern oncology.

The present review primarily focuses on CAT-VTE, given its substantially higher incidence and clinical relevance in patients with cancer. Arterial thrombotic complications are discussed only where specifically relevant.

## 2. Materials and Methods

This narrative review was conducted through a comprehensive literature search of the PubMed/MEDLINE, Scopus, and Web of Science databases up to April 2026. The following keywords and Medical Subject Heading (MeSH) terms were used in different combinations: “cancer-associated thrombosis”, “venous thromboembolism”, “breast cancer”, “ovarian cancer”, “endometrial cancer”, “hormones”, “endocrine therapy”, “tamoxifen”, “hypercoagulability”, “thromboprophylaxis”, and “immune-thrombosis”. Priority was given to recent clinical guidelines, meta-analyses, translational studies, randomized clinical trials, and high-impact reviews published in English. Additional references were identified through manual screening of reference lists from selected articles. Given the narrative, non-systematic design of the present review, a formal PRISMA-based study-selection process was not applied; nevertheless, the literature search strategy and source-selection approach are reported to improve transparency and reproducibility.

## 3. Pathophysiology of CAT in Gynecological Cancers: The Role of Hormones in Modulating Hemostasis

Sex hormones exert complex regulatory effects on hemostasis and vascular homeostasis [12]. Estrogens are the hormones most strongly associated with thrombosis and promote hypercoagulability through increased hepatic synthesis of coagulation factors II, VII, VIII, X, and fibrinogen, together with reduced anticoagulant activity mediated by protein S depletion and acquired resistance to activated protein C [12]. Additionally, estrogens influence endothelial biology by modulating nitric oxide production, vascular permeability, inflammatory signaling, and platelet activation [13]. These mechanisms partly explain the increased thrombotic risk observed during oral contraceptive use, menopausal hormone replacement therapy, pregnancy, and endocrine treatment in hormone-sensitive cancers [14]. Although progesterone has been shown to exert minimal direct effects on coagulation pathways, its presence within combined hormonal regimens may facilitate vascular inflammation and endothelial alterations, potentially influencing thrombotic risk [15]. In oncologic patients, endocrine influences interact synergistically with tumor-driven coagulation activation. Tumor cells release Tissue Factor (TF)-bearing extracellular vesicles capable of activating factor VII and initiating thrombin generation [16]. Inflammatory cytokines such as interleukin-6 (IL-6) and tumor necrosis factor-alpha (TNF-α) amplify endothelial activation and platelet aggregation [17]. NETs (Neutrophil Extracellular Traps) further contribute to immune-thrombosis by promoting platelet adhesion, fibrin deposition, and endothelial injury [18].

**Figure 1:**
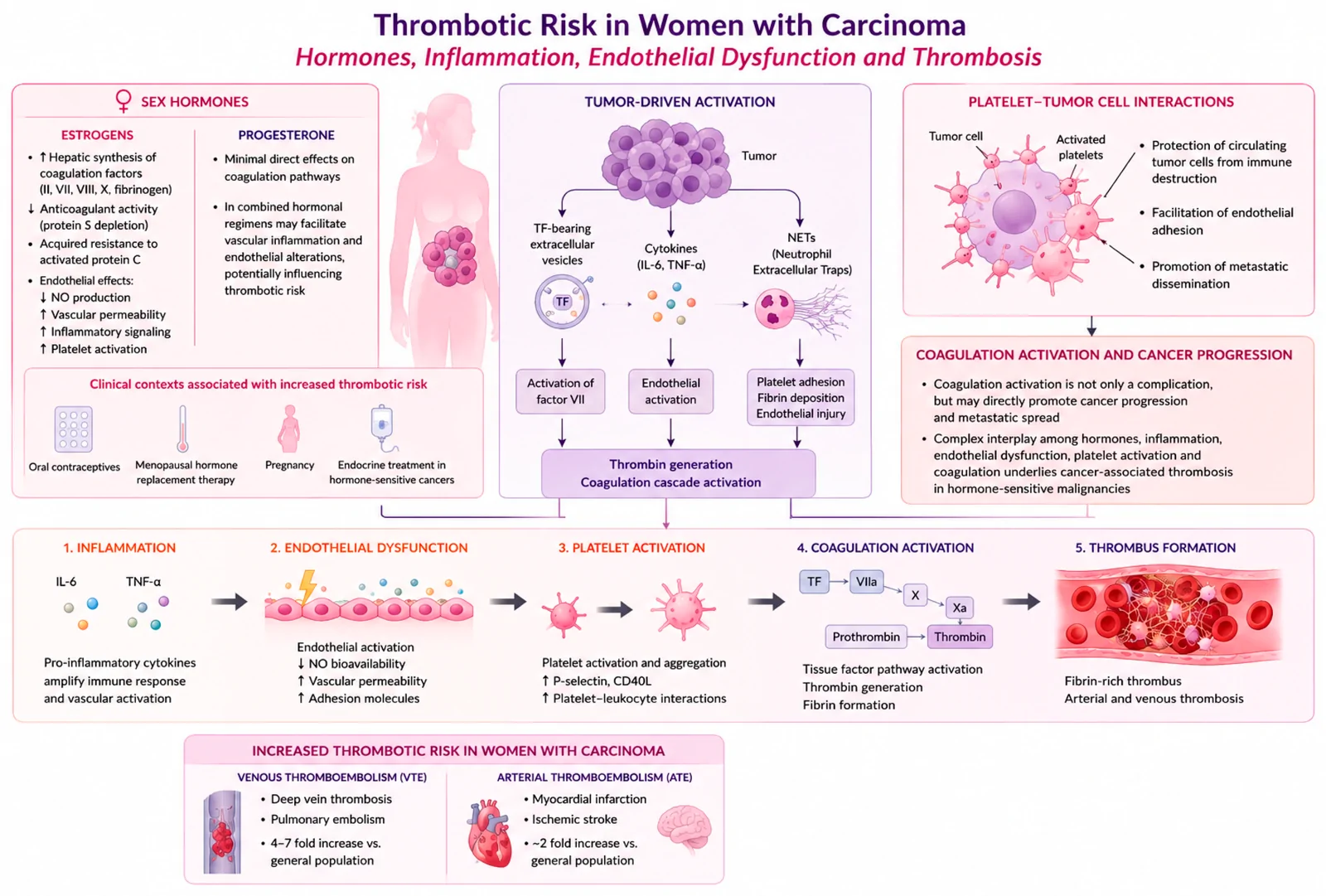
Mechanism of cancer-associated thrombosis. Schematic representation of tumor-induced activation of coagulation pathways, endothelial dysfunction, and platelet activation leading to thrombus formation in women with gynecological cancers. Arrows indicate an increase (↑) or decrease (↓).

Figure 1 illustrates the interaction between hormone inflammation, endothelial dysfunction, and thrombosis in cancer.

Increasing evidence suggests that NET-mediated coagulation activation represents a central mechanism linking inflammation and thrombosis in cancer patients. Another important mechanism involves platelet–tumor cell interactions. Activated platelets protect circulating tumor cells from immune-mediated destruction, facilitate endothelial adhesion, and promote metastatic dissemination [19]. Beyond immune evasion, platelet-derived signals and platelet–tumor cell crosstalk further enhance tumor cell survival, vascular arrest, and extravasation, thereby actively contributing to metastatic spread [20]. In parallel, activation of the coagulation cascade—particularly through tissue factor-driven pathways—promotes not only thrombin generation but also pro-angiogenic and pro-invasive signaling within the tumor microenvironment, including VEGF upregulation and PAR-mediated effects, indicating that coagulation activation is not merely a complication of malignancy but may directly contribute to cancer progression [21]. This intricate interplay among hormones, inflammation, endothelial dysfunction, platelet activation, and coagulation represents the biological basis of CAT in hormone-sensitive malignancies.

## 4. Breast Cancer and Venous Thromboembolism

Breast cancer is the most frequently diagnosed malignancy among women worldwide, accounting for approximately 2.3 million new cases annually [22]. Although breast cancer is generally associated with lower intrinsic thrombotic risk compared with ovarian cancer, its extremely high incidence and prolonged exposure to systemic therapies make it one of the most important contributors to CAT in women [23]. Approximately 70% of breast cancers express estrogen and/or progesterone receptors, making endocrine therapy a cornerstone of treatment [24]. Thrombotic risk in breast cancer is multifactorial and influenced by patient-related, tumor-related, and treatment-related factors. Advanced age, obesity, diabetes, cardiovascular disease, reduced mobility, and metastatic disease significantly increase VTE risk [25]. Obesity contributes to chronic inflammation, endothelial dysfunction, and increased circulating procoagulant factors, further amplifying hypercoagulability [26]. In population-based cohorts of patients with breast cancer, the incidence of VTE was estimated at approximately 5 per 1000 person-years (0.5% per year) [5]. Beyond VTE, breast cancer patients may also experience arterial thrombotic complications, including myocardial infarction and ischemic stroke, particularly during exposure to cardiotoxic therapies, such as anti-HER2 therapies [27].

Among treatment-related factors, endocrine therapy represents one of the most important determinants of thrombotic risk. Tamoxifen, a selective estrogen receptor modulator (SERM), is associated with a two- to three-fold increased risk of VTE compared to age-matched controls [28]. Mechanistically, tamoxifen exerts partial estrogen agonist effects on hepatic coagulation pathways, increasing thrombin generation and reducing endogenous anticoagulant activity [6]. Conversely, aromatase inhibitors appear to have a significantly safer thrombotic profile, as they reduce systemic estrogen levels without inducing the procoagulant changes associated with tamoxifen [6]. Chemotherapy further increases thrombotic risk through endothelial injury, cytokine release, platelet activation, and central venous catheter use [29]. Population-based studies demonstrate a marked increase in the incidence of VTE during the first months of chemotherapy administration, with a 2- to 4-fold higher risk compared with baseline [30]. The introduction of CDK4/6 inhibitors has added additional complexity to thrombotic risk assessment. Emerging evidence suggests increased VTE rates among patients receiving palbociclib, ribociclib, or abemaciclib in combination with endocrine therapy, with incidences ranging from 1–5% in pivotal clinical trials during follow-up, while real-world studies have reported cumulative incidence rates approaching 10% within the first year of treatment [31,32]. Patients with metastatic breast cancer exhibit substantially higher thrombotic burden compared with those affected by early-stage disease, with a 6-fold higher risk [33]. Advanced tumor burden promotes systemic inflammation, endothelial dysfunction, prolonged immobility, and increased release of procoagulant extracellular vesicles. In contrast to conventional chemotherapy, anti-HER2 therapies are not associated with an increased risk of VTE; their toxicity profile is primarily defined by cardiotoxicity, whereas trastuzumab emtansine has been linked to clinically relevant thrombocytopenia, including grade ≥3 thrombocytopenia (platelet count 25,000–50,000/mm^3^), warranting careful monitoring of hematologic parameters [27,34].

Breast cancer surgery is generally associated with a low 30-day postoperative VTE incidence (approximately 0.2–1.2%) in oncologic series under perioperative thromboprophylaxis [34]; however, prolonged surgical procedures, reconstructive surgery, obesity, and advanced age (>70 yeas) significantly increase thrombotic risk [35]. Accordingly, pharmacologic thromboprophylaxis should be considered selectively in patients presenting these additional risk factors. Overall, individualized thrombotic risk assessment is essential in breast cancer patients to appropriately balance thrombosis prevention and bleeding risk.

## 5. Endometrial Cancer

Endometrial cancer is closely associated with obesity, metabolic syndrome, insulin resistance, and prolonged exposure to unopposed estrogens, all of which contribute to thrombotic risk [36]. The reported incidence of VTE varies widely, ranging from 1.5% in localized disease to 10.5% in advanced-stage cancer, depending on study design and follow-up duration [37]. Among the underlying mechanisms, obesity represents one of the most relevant shared pathogenic factors linking endometrial cancer and thrombosis. Adipose tissue promotes chronic low-grade inflammation, endothelial dysfunction, increased PAI-1 levels, and coagulation activation [38]. Visceral obesity additionally acts as an active endocrine organ capable of releasing adipokines, leptin, resistin, and proinflammatory cytokines, all contributing to endothelial dysfunction and impaired fibrinolysis [39,40]. Patients with endometrial cancer frequently present with cardiovascular comorbidities, diabetes, hypertension, and advanced age, further amplifying VTE susceptibility [41]. In addition, the recent molecular classification of endometrial cancer has provided new insights into biological heterogeneity that may also have implications for thrombotic risk. Aggressive molecular subtypes, particularly p53-abnormal tumors, are frequently associated with higher inflammatory activity and advanced-stage presentation, potentially contributing to increased coagulation activation [42].

Mismatch repair-deficient tumors may additionally exhibit immune-mediated inflammatory activation capable of amplifying endothelial dysfunction and thrombogenesis [43]. Surgical management often requires extensive pelvic procedures and lymphadenectomy, exposing patients to substantial perioperative thrombotic risk [44]. The incidence of postoperative VTE within 30 days of gynecologic cancer surgery is estimated at approximately 2–4% in patients with endometrial cancer, despite the perioperative thromboprophylaxis, and remains higher than the rates generally reported after breast cancer surgery [39]. Radiotherapy and chemotherapy may further exacerbate endothelial injury and coagulation activation, particularly in advanced-stage disease [45]. Extended postoperative thromboprophylaxis for up to 28 days is currently recommended in high-risk patients undergoing major gynecologic oncologic surgery, particularly after open abdominal or pelvic procedures, as these individuals are at especially elevated risk of VTE events [44,46]. In a large cohort of women with endometrial cancer, the cumulative incidence of VTE was 2.7% within 24 months of diagnosis, and major surgery was independently associated with an increased risk of VTE (HR 1.7, 95% CI 1.1–2.6). Notably, 26.4% of all VTE events occurred within 3 months following major pelvic or abdominal cancer surgery [37].

## 6. Ovarian Cancer

Ovarian cancer is among the most thrombogenic solid malignancies, with reported VTE incidence ranging between 11% and 27% across heterogeneous study populations and follow-up durations [47]. Several studies have demonstrated that ovarian cancer carries substantially higher thrombotic risk compared with breast and endometrial cancers [48]. In addition, patients with ovarian cancer experience the highest incidence of recurrent VTE while receiving anticoagulant treatment, with rates reported up to 7.29 events per 100 patient-years [47].

The marked thrombogenicity of ovarian cancer reflects the interaction among aggressive tumor biology, extensive tumor burden, inflammatory activation, and intensive multimodal treatment strategies.

Tumor cells express high levels of TF (tissue factor), which directly activates the coagulation cascade and promotes thrombin generation [49]. Elevated levels of inflammatory cytokines, including IL-6 and TNF-α, further contribute to endothelial dysfunction and systemic hypercoagulability [50]. NET formation also appears to play a major role in ovarian cancer-associated immune-thrombosis [18]. Histologic subtype significantly influences thrombotic risk. Ovarian clear cell carcinoma (OCCC) demonstrates particularly high VTE incidence because of elevated TF expression, inflammatory activation, and hypoxia-related signaling pathways [51]. Several studies have reported VTE rates exceeding 30% in patients with advanced OCCC [29]. Advanced-stage disease, ascites, peritoneal carcinomatosis, and bulky pelvic masses additionally increase thrombotic risk through venous compression, immobility, and chronic inflammation [52]. Anatomical factors, including primary tumor or lymph node external compression, may further increase the risk of thrombosis at unusual sites, such as the splanchnic veins [49,53].

Treatment-related factors substantially contribute to thrombotic burden. Platinum-based chemotherapy has been associated with nearly seven-fold increased VTE risk [54]. Major debulking procedures together with hyperthermic intraperitoneal chemotherapy (HIPEC) can trigger a pronounced systemic inflammatory reaction and cause significant disruption of endothelial homeostasis [55]. Targeted therapies such as anti-VEGF agents and PARP inhibitors (poly ADP ribose polymerase) may also modestly increase thrombotic risk [4,56]. Antiangiogenic drugs, particularly, have been associated with both venous and arterial thromboembolic complications. Given the extremely high thrombotic burden observed in ovarian cancer, current international guidelines strongly recommend extended postoperative thromboprophylaxis following major pelvic surgery, up to 28 days [44,46].

**Figure 2:**
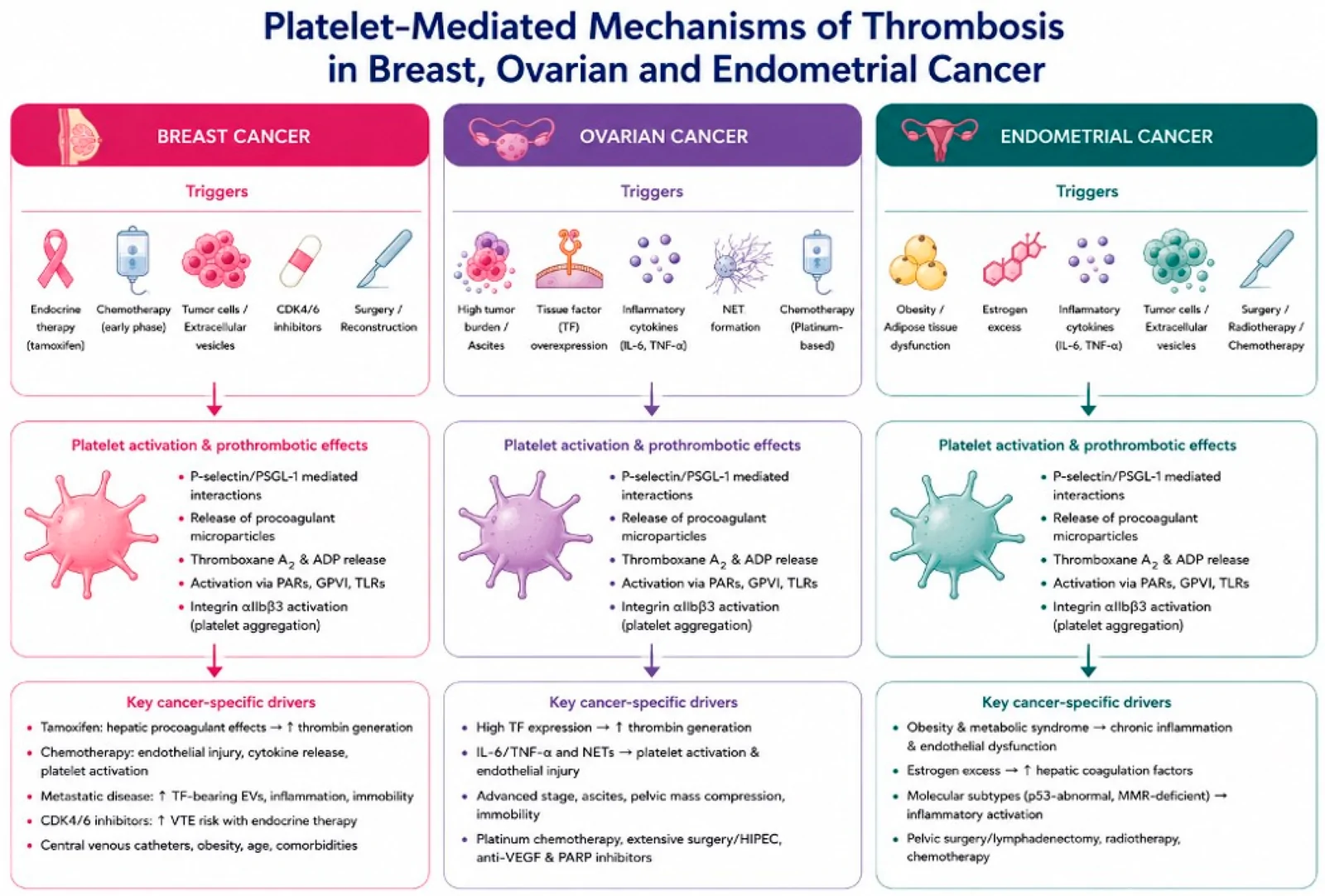
Schematic representation of venous thromboembolism risk and contributing factors in breast, ovarian, and endometrial cancer. Arrows (↑) indicate an increase.

Differences in VTE incidence and associated risk factors across breast, endometrial, and ovarian cancer are detailed in Table 1 and Figure 2.

## 7. Biomarkers of Cancer-Associated Thrombosis

Several biomarkers have been investigated for their potential role in predicting thrombotic risk in oncologic patients. Elevated D-dimer levels are consistently associated with increased VTE risk and poor oncologic outcomes; however, it should be noted that D-dimer is frequently elevated in patients with cancer even in the absence of thrombosis, reflecting underlying malignancy-related inflammation, tumor burden, and comorbid conditions. Therefore, outside of well-designed studies with carefully selected populations, its standalone clinical utility remains limited and its implementation in real-world settings is still not fully established [57,58]. Similarly, soluble P-selectin (sP-selectin), a circulating glycoprotein shed from activated platelets and endothelial cells, has demonstrated predictive value for the development of venous thromboembolism (VTE) in ambulatory cancer patients [2], although its use is constrained by assay availability and lack of standardization across laboratories, which limits its applicability in routine practice and real-world populations. Circulating TF-positive extracellular vesicles represent promising biomarkers of hypercoagulability, particularly in ovarian cancer [59]. Inflammatory markers such as IL-6, TNF-α, and C-reactive protein have also demonstrated associations with CAT development [60,61]. Despite encouraging evidence, biomarker-guided thromboprophylaxis has not yet been fully integrated into routine clinical practice because of limited standardization, heterogeneous predictive performance across tumor types, and restricted availability of assays in clinical laboratories. Future strategies will likely combine clinical variables with molecular and inflammatory biomarkers to improve individualized thrombotic risk prediction.

## 8. Risk Stratification and Personalized Thromboprophylaxis

Risk assessment models (RAMs) play an increasingly important role in identifying cancer patients who may benefit from primary thromboprophylaxis. Among these, the Khorana score remains the most widely used tool in oncology for stratifying thrombotic risk and identifying high-risk individuals (score ≥ 2) who may benefit from primary thromboprophylaxis [62]. However, its predictive performance in gynecologic and breast malignancies remains suboptimal, particularly in ambulatory patients receiving systemic therapies [63]. Patient education regarding CAT, including symptoms, risk factors, and preventive measures, is also strongly recommended. Current ASCO (American Society of Clinical Oncology), ESMO (European Society for Medical Oncology), ESC (European Society of Cardiology) and ASH (American Society of Hematology) guidelines recommend considering primary thromboprophylaxis in ambulatory high-risk patients receiving systemic therapy, particularly those with advanced ovarian cancer, metastatic disease, previous VTE, obesity, or multiple thrombotic risk factors [33,44,46,64].

In the ambulatory setting, low-molecular-weight heparin (LMWH) remains a standard option for primary thromboprophylaxis, while direct oral anticoagulants (DOACs), including apixaban and rivaroxaban, represent effective alternatives in selected high-risk patients receiving systemic therapy, with treatment generally corresponding to the long-term phase of cancer-associated thrombosis (approximately 3–6 months in clinical studies), with duration thereafter individualized according to thrombotic and bleeding risk and the persistence of active cancer or systemic therapy [46,65]. Evidence indicates that DOAC prophylaxis is associated with a reduction in VTE incidence compared with placebo, although this benefit is accompanied by a modest increase in bleeding risk [64]; therefore, careful bleeding risk assessment is essential, particularly in patients with gynecologic malignancies, mucosal involvement, thrombocytopenia, renal impairment, or recent surgery [66]. ESC guidelines further emphasize a structured TBIP approach (thrombosis, bleeding, interactions, patient preferences) and the concept of a “double-risk profile” characterized by simultaneous thrombotic and hemorrhagic risk in cancer patients [33,67,68].

Shared decision-making should also account for potential drug–drug interactions, hepatic and renal function, and patient preferences. Attention should be paid to CYP3A4 and P-glycoprotein-mediated interactions between DOACs and anticancer or supportive therapies, as these pathways are frequently modulated by anticancer agents and concomitant medications in oncology patients, potentially leading to clinically relevant increases in bleeding risk or reductions in anticoagulant efficacy [33].

In the surgical setting, all patients undergoing oncologic procedures should undergo systematic preoperative assessment of both thrombotic and bleeding risk, integrating patient-related factors, validated RAM score, and procedural characteristics, including type and duration of surgery [46,69]. Pharmacologic thromboprophylaxis with LMWH is recommended as standard of care in patients undergoing major cancer surgery without high bleeding risk, with unfractionated heparin (UFH) as an alternative option, and should be continued for at least 10 days [46]. Guidelines further support a preference for LMWH over UFH in this setting, given a lower risk of heparin-induced thrombocytopenia and reduced wound hematoma rates [33].

Mechanical methods, such as intermittent pneumatic compression or graduated compression stockings, may be used when pharmacologic prophylaxis is contraindicated and can be combined with anticoagulation in very high-risk patients [33,46].

Extended postoperative thromboprophylaxis with LMWH for up to 28 days is recommended after major abdominal or pelvic oncologic surgery, including gynecologic malignancies, as a substantial proportion of VTE events occur after hospital discharge [70]. This strategy has shown benefit in both open and selected minimally invasive procedures [44,46].

Although platelet activation plays an important role in cancer-associated thrombosis, antiplatelet agents such as aspirin and P2Y12 receptor inhibitors are not currently recommended as substitutes for anticoagulant thromboprophylaxis for the primary prevention of cancer-associated venous thromboembolism in patients with breast, ovarian, or endometrial cancer. Their use in patients with cancer is generally driven by established cardiovascular or cerebrovascular indications, including acute coronary syndromes, percutaneous coronary intervention, and selected ischemic cerebrovascular events, rather than by malignancy itself. When managing patients who require concurrent anticoagulation for cancer-associated VTE and antiplatelet therapy for an arterial indication, treatment must weigh the heightened bleeding risk against the threat of thrombosis, tailoring decision-making to the patient’s individual baseline risk, underlying cancer, and active cancer therapies. In patients requiring dual antiplatelet therapy after coronary intervention, contemporary cardio-oncology guidance favors the shortest appropriate duration and generally prefers aspirin plus clopidogrel over more potent P2Y12 inhibitors in patients with cancer. Thus, despite the biological relevance of platelets in CAT, current evidence does not support routine aspirin or P2Y12 inhibition specifically for VTE prevention in gynecological or breast malignancies [7,33,71].

A summary of anticoagulant thromboprophylaxis indications across breast, ovarian, and endometrial cancer is provided in Table 2.

**Figure 3:**
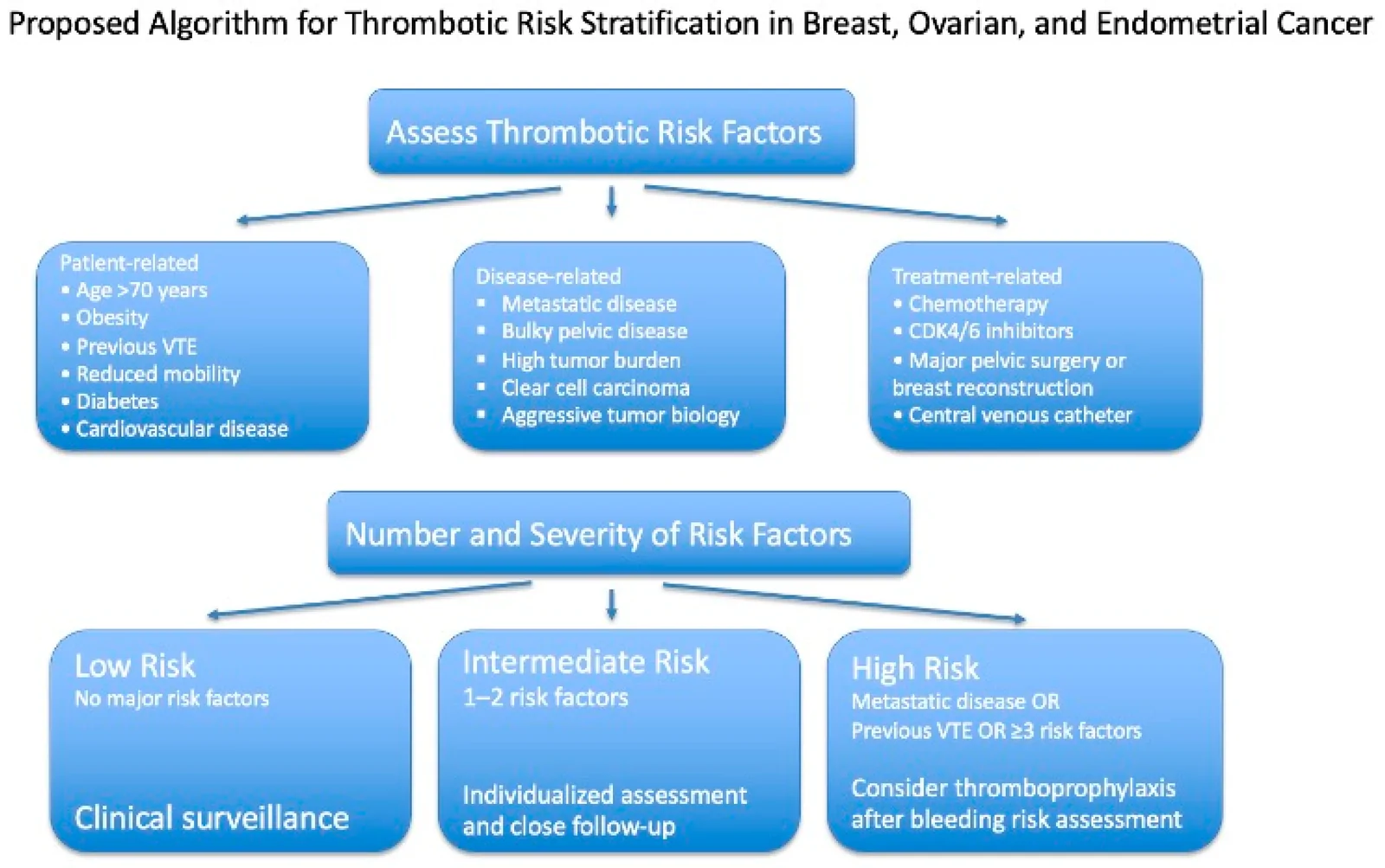
Proposed algorithm for thrombotic risk stratification in patients with breast cancer.

A practical approach to thrombotic risk stratification in breast cancer patients is proposed in Figure 3.

## 9. Future Perspectives

Future thrombosis prevention strategies will likely rely on precision medicine approaches integrating clinical variables, tumor biology, inflammatory and endocrine profiles, and artificial intelligence-based predictive models. Emerging evidence suggests that immunothrombosis and NET-mediated coagulation activation may represent promising therapeutic targets in cancer-associated hypercoagulability. In parallel, liquid biopsy technologies and circulating biomarker profiling may further improve individualized thrombotic risk prediction and allow for dynamic monitoring of hypercoagulability during systemic anticancer treatment. Recent studies have suggested that circulating tumor DNA (ctDNA) and cell-free DNA (cfDNA) levels are associated with cancer-associated VTE and may provide additional prognostic information beyond conventional clinical risk scores. In particular, ctDNA-based approaches have shown independent associations with VTE risk and may improve discrimination when combined with clinical variables [72]; however, the incremental predictive value remains modest, and biomarker-based and machine-learning prediction models are still under investigation and require prospective external validation before routine clinical implementation [73]. A deeper understanding of sex-specific coagulation mechanisms may also contribute to the development of tailored thromboprophylaxis strategies in hormone-sensitive malignancies, ultimately improving the balance between thrombosis prevention and bleeding risk.

## 10. Conclusions

CAT remains a major challenge in breast and gynecologic oncology. Breast, ovarian, and endometrial cancers exhibit distinct thrombotic phenotypes shaped by endocrine signaling, tumor biology, systemic inflammation, obesity, and anticancer therapies. Breast cancer is characterized predominantly by endocrine therapy-related thrombotic risk, particularly with tamoxifen exposure. Ovarian cancer represents one of the most thrombogenic solid malignancies because of aggressive tumor biology, extensive inflammatory activation, and intensive multimodal treatment strategies. Endometrial cancer demonstrates a strong association with obesity-driven inflammation, metabolic dysfunction, and perioperative thrombosis. Recognition of sex-specific mechanisms underlying CAT is essential to optimize risk stratification and personalize thromboprophylaxis strategies. As oncologic outcomes continue to improve, prevention of thrombotic complications will become an increasingly important component of survivorship-oriented cancer care. Future advances in biomarker research, molecular profiling, and artificial intelligence-based prediction models may further refine individualized thromboprophylaxis and improve outcomes in women with hormone-sensitive malignancies.

## Figures and Tables

**Table 1:** Summary of venous thromboembolism risk and contributing factors in breast, ovarian, and endometrial cancer.

Cancer Type	Overall VTE Risk	Main Biological Drivers	Clinical Features Associated with Increased VTE Risk	Implications for Thromboprophylaxis
**Breast cancer**	**Intermediate** (≈0.5%/year; higher in metastatic disease) [5,27,28,31,32,33,34,35]	Estrogen signaling, endocrine therapy (tamoxifen), platelet activation [6,28,29]	Metastatic disease, obesity, advanced age, chemotherapy, CDK4/6 inhibitors, prolonged surgery, central venous catheter [25,27,30,31,32,33,34,35]	Routine prophylaxis is **not recommended** in ambulatory patients; consider individualized prophylaxis in high-risk patients. Perioperative prophylaxis should be reserved for patients undergoing major surgery or presenting additional risk factors [4,33,46].
**Endometrial cancer**	**Intermediate–High** (1.5–10.5%) [37]	Obesity-related inflammation, metabolic dysfunction, prolonged estrogen exposure, molecular high-risk subtypes [38,39,40,41,42,43]	Obesity, metabolic syndrome, advanced age, p53-abnormal tumors, major pelvic surgery with lymphadenectomy, chemotherapy, radiotherapy [37,39,41,44,45]	Thromboprophylaxis should be guided by individual risk assessment. Extended postoperative prophylaxis is recommended after major abdominal or pelvic surgery in high-risk patients [44,46].
**Ovarian cancer**	**Very high** (11–27%; >30% in clear-cell carcinoma) [29,47,48]	Tissue factor overexpression, systemic inflammation, NET formation, aggressive tumor biology [18,49,50,51]	Advanced stage, clear-cell histology, bulky pelvic disease, ascites, platinum-based chemotherapy, cytoreductive surgery ± HIPEC, anti-VEGF therapy [29,52,53,54,55,56]	Extended postoperative thromboprophylaxis (up to 28 days) is recommended after major surgery. Primary prophylaxis should be strongly considered in high-risk ambulatory patients receiving systemic therapy [4,44,46].

Abbreviations: CDK4/6, cyclin-dependent kinases 4 and 6; HIPEC, hyperthermic intraperitoneal chemotherapy; NET, neutrophil extracellular traps; VEGF, vascular endothelial growth factor.

**Table 2:** Guideline-based recommendations for thromboprophylaxis in breast, ovarian, and endometrial cancer.

Clinical Setting	Breast Cancer	Endometrial Cancer	Ovarian Cancer
**Ambulatory patients receiving systemic therapy**	Routine thromboprophylaxis is **not recommended**. Consider LMWH or DOACs only in selected high-risk patients after individual risk assessment [4,32,46].	Consider thromboprophylaxis only in selected high-risk patients after individualized assessment of thrombotic and bleeding risk [4,32,45,46].	Primary thromboprophylaxis should be considered in high-risk patients with low bleeding risk; LMWH or DOACs may be used [4,32,45,46].
**Major cancer surgery**	Pharmacological thromboprophylaxis is recommended according to surgical and patient-related risk; LMWH is the preferred agent [4,32,46].	Pharmacological thromboprophylaxis with LMWH is recommended in patients undergoing major abdominal or pelvic surgery [32,45,46].	Pharmacological thromboprophylaxis with LMWH is recommended in all patients undergoing major surgery [32,45,46].
**Extended postoperative prophylaxis**	Not routinely indicated; consider only in selected high-risk patients [32,46].	Extended LMWH prophylaxis for **28 days** after major abdominal or pelvic surgery is recommended [32,45,46].	Extended LMWH prophylaxis for **28 days** after major abdominal or pelvic surgery is recommended [32,45,46].
**Preferred anticoagulant strategy**	LMWH for surgical prophylaxis; DOACs may be considered in selected ambulatory patients [4,46].	LMWH is preferred after surgery; DOACs may be considered in selected ambulatory patients [32,46].	LMWH remains the preferred agent after surgery; DOACs are an option in selected ambulatory patients [32,46].

Abbreviations: DOACs, direct oral anticoagulants; LMWH, low-molecular-weight heparin.

## Data Availability

The original contributions presented in the study are included in the article. Further inquiries can be directed to the corresponding author.

## Abbreviations

The following abbreviations are used in this manuscript:

VEGF	Vascular Endothelial Growth Factor
CAT	Cancer-associated thrombosis
VTE	Venous Thromboembolism
CDK4/6	Cyclin-Dependent Kinases 4 and 6
OCCC	Ovarian Clear Cell Carcinoma
TF	Tissue Factor
IL-6	Interleukin-6
TNF-α	Tumor Necrosis Factor Alpha
NET	Neutrophil Extracellular Trap
HIPEC	Hyperthermic Intraperitoneal Chemotherapy
MMR	Mismatch Repair
RAMs	Risk assessment models
